# A Method for Sensor-Based Activity Recognition in Missing Data Scenario

**DOI:** 10.3390/s20143811

**Published:** 2020-07-08

**Authors:** Tahera Hossain, Md. Atiqur Rahman Ahad, Sozo Inoue

**Affiliations:** 1 Department of Applied Science for Integrated System Engineering, Kyushu Institute of Technology, Kitakyushu 804-8550, Japan; 2 Department of Media Intelligent, Osaka University, Ibaraki 567-0047, Japan; atiqahad@du.ac.bd; 3 Department of Electrical and Electronic Engineering, University of Dhaka, Dhaka 1000, Bangladesh; 4 Department of Human Intelligence Systems, Kyushu Institute of Technology, Kitakyushu 808-0196, Japan; sozo@brain.kyutech.ac.jp

**Keywords:** human activity recognition (HAR), sensor network, missing values, random forest, SVM

## Abstract

Sensor-based human activity recognition has various applications in the arena of healthcare, elderly smart-home, sports, etc. There are numerous works in this field—to recognize various human activities from sensor data. However, those works are based on data patterns that are clean data and have almost no missing data, which is a genuine concern for real-life healthcare centers. Therefore, to address this problem, we explored the sensor-based activity recognition when some partial data were lost in a random pattern. In this paper, we propose a novel method to improve activity recognition while having missing data without any data recovery. For the missing data pattern, we considered data to be missing in a random pattern, which is a realistic missing pattern for sensor data collection. Initially, we created different percentages of random missing data only in the test data, while the training was performed on good quality data. In our proposed approach, we explicitly induce different percentages of missing data randomly in the raw sensor data to train the model with missing data. Learning with missing data reinforces the model to regulate missing data during the classification of various activities that have missing data in the test module. This approach demonstrates the plausibility of the machine learning model, as it can learn and predict from an identical domain. We exploited several time-series statistical features to extricate better features in order to comprehend various human activities. We explored both support vector machine and random forest as machine learning models for activity classification. We developed a synthetic dataset to empirically evaluate the performance and show that the method can effectively improve the recognition accuracy from 80.8% to 97.5%. Afterward, we tested our approach with activities from two challenging benchmark datasets: the human activity sensing consortium (HASC) dataset and single chest-mounted accelerometer dataset. We examined the method for different missing percentages, varied window sizes, and diverse window sliding widths. Our explorations demonstrated improved recognition performances even in the presence of missing data. The achieved results provide persuasive findings on sensor-based activity recognition in the presence of missing data.

## 1. Introduction

Human activity recognition (HAR), using sensor-based systems, is one of the most prominent fields of research. One of the primary goals of HAR is to understand the daily behaviors of people through the interpretation of information from different sensors collected from people and their surrounding living environments. Sensor-based human activity recognition has a significant impact on healthcare monitoring, assisted-living, surveillance, entertainment, etc. [1]. One of the main reasons for the exploration of HAR-related research is the increase in elderly population all over the world [2,3]. The monitoring of elderly people’s daily activity becomes crucial to improve their quality of life and to prevent any sudden accidents, such as falls [4,5]. Through advanced technology, it is possible to detect human body movement. There are lots of works on video-based action or activity recognition [6,7,8,9]; however, video-based methods have privacy concerns that deter many users from video-based systems, and on the contrary, encourage users to adopt sensor-based activity analysis. Apart from the privacy issue, video-based activity recognition has issues with occlusions, segmenting multiple people in the scenes, etc. [6,8,9]. Many researchers explored sensor-based activity recognition techniques [10,11]. In this scheme, wearable sensor data were collected from each individual. Afterward, through near-by access points, data were transmitted to servers. Usually, various features are extracted from these time-series continuous data. Finally, machine learning approaches were explored for recognition of different activities [7].

Researchers are also conscious of real-time applications for hospitals and nursing homes [12,13]; however, data collection in a controlled-environment or in the wild has challenges [14]. A real field study was introduced in [15] to facilitate nursing home activity logs and activity recognition. This experiment in nursing care facilities introduce real life activity recognition challenges from field data. Data were collected for four months from a nursing care center. The data included nursing care records at nursing care facilities, and data from activity labels and sensors obtained through smartphones. Evaluation of the data showed a lower recognition accuracy in the real-life situation [15]; however, a controlled experiment using motion capture and smartphone sensors have demonstrated that it is possible to recognize nursing activities by means of motion sensing with higher accuracy [16]. One limitation for the recognition of complex activities in the real field is the use of a single inertial sensor in the phone. Moreover, recognition of regular activity and complex activity from a real-life situation is challenging. Along with these issues, data loss or missing data are a crucial concern while collecting data in the wild. Usually, human walking, jogging, running, sitting, standing, stair-up, stair-down, cooking activities, sports activities, etc. are recognized from standard sensors without any corrupted or missing data [1,8,17]. There are more than 150 datasets for the sensor-based activity arena [1,17]. Usually, these datasets obtain continuous data from the accelerometer or other sensors, and as such, there is no issue of missing data [17].

In a real-life scenario, however, missing data are prevalent in any wireless sensor networks or body-area wireless sensor networks [18]. Data are lost or the presence of incomplete data can be due to various reasons. A few prominent reasons for data missing are: distance within sensors and nearest access point, sensor malfunction, lack of sufficient power in the battery, collision of data packets, transmission problems, dropped connections, troubles in sensor synchronization, corrupted readings, weak Wi-Fi signal strength, limited network coverage, and so on [18,19,20,21,22,23,24]. Mobile devices have limited memory capacity and computational ability that may cause data to be missed [25].

There are three categories or patterns of missing data. Usually, data can be missed at any random order at any period (i.e., missing in random order (MAR)), or data can be missed in a periodic manner (i.e., data not missing in a random order), or the third pattern is the case where data are completely missed or lost, in any random manner [18]. The latter pattern occurs mainly when the battery is off, or when the system has technical problems. It can be predicted as data missing entirely.

The random missing pattern is more realistic because, in real nursing care or a healthcare center, staff use wearable sensors (e.g., accelerometer and gyroscope) for each individual. These sensors’ data are then transferred to near-by access points and important data are sent to a server using a wireless sensor network. In some cases, sensing, computing, and communications can be performed on a single chip, which reduce the cost and facilitate the deployment in even larger numbers. Each node can sense its local environment, perform necessary computations and processing, and send the sensory data through multi-hop nodes. Due to these computational complexities, sensors data can be continuously received, and sometimes be missed without following any patterns. In the real field, time-series sensor data are not continuously missed, or not missed by following a specific pattern, or not missed having a rhythmic period.

During the missing pattern identification, distinguishing a complete missing pattern is relatively easier; however, in real-life data collection, the most common missing pattern is missing at random. The random missing pattern is difficult to decipher. Researchers have been engaged on various data imputation techniques, which are not explored in our work [21,26]. The main contributions of this research are highlighted below: In this work, missing data were not recovered or reconstructed. Instead, human activity recognition performance having missing data was explored based on the most realistic scenario in wireless sensor network. Without exploring any specific data imputation technique, through this approach, any overhead related to data imputation processing time and storage can be reduced. We propose an approach where activity recognition accuracy can be enhanced while having missing data in the dataset. Based on our study, there is no work considering the missing at random pattern without data imputation for sensor-based activity recognition in the manner that we consider in this paper. Two challenging benchmark datasets were explored to justify the approach.

For this study, we generated various random missing percentages (2%, 3%, 5%, 7%, 8%, and 10%) on two benchmark datasets. We unearthed different statistical features from the time-series data for classification. To engulf the entire observation of the dataset, an overlapping windowing technique was followed. In the original dataset, there was no missing information. We refer to these data as ’clean data’. Afterward, we created different percentages of randomly-missing datasets for evaluation. In the recognition process, we classified the action instances in three scenarios: (i) when both training dataset and testing dataset were clean; (ii) when training dataset was clean but the testing dataset had missing data; (iii) when both training and testing dataset had missing data. In this paper, we explored two challenging benchmark datasets, the human activity sensing consortium (HASC) dataset [27] and the Single Chest dataset [28] for the experimental proofs.

The paper is organized as follows: In Section 1, we introduce the background. Afterward, we enlist some related works in Section 2. The workflow of our method is presented in Section 3. Later sections cover the experimental results and discussion respectively. Finally, the concluding remarks are made in Section 6.

## 2. Related Works

In recent years, there have been numerous research works in the field of activity recognition using machine learning and deep learning approaches. Data can be collected through a video-based platform or a wearable sensor-based system. Occlusion, privacy issues, huge data, huge computational costs, etc. are some main obstacles to use a video-based system for an elderly care center or assisted living center for monitoring elderly people’s regular activity [6]. On the other hand, in recent years, wearable sensors have attained lots of attention in the research community.

Through accelerometers and gyro sensors, it becomes easier to detect and recognize various human movements. In this regard, smartphones play a critical role in contributing in this arena. In a smartphone, various sensors are already mounted, such as a three-axis acceleration sensor that changes the data based on different movements, and an angular velocity sensor (also called a gyro sensor) that detects the angle (posture), angular velocity, or angular acceleration of an object. Because of continuous monitoring and collecting data, there is a significant amount of sensor data that carries important information for analysis. Researchers are facing a number of challenges for proper data collection, data annotation, misclassification, etc. related to this field.

Activity recognition in a real nursing care center is different and more difficult than in a controlled lab environment. The authors in [14] addressed some challenges for a real nursing care center experiment. Data annotation, proper timestamp, and loss of important data are crucial issues during data collection in a real environment [14,29]. The authors of [30] proposed an approach to support energy-efficient data collection. Their compression-based approach can balance the data quality and data transmission overheads. Through this method, the authors have proposed an energy-efficient uninterrupted smartphone sensor-based activity recognition approach. In a smartphone-based activity recognition system, the energy constraint is an important issue to be addressed for continuous sensing applications. It causes data missing issues, which are unavoidable due to the nature of storage of proper power sources.

Apart from smartphones, the wireless sensor network (WSN) has attracted lots of attention from academia in healthcare and activity recognition [31]. WSN has been used for sensor data communication through various sensors. In WSN, missing data is a recurrent and challenging issue [21,24]. In WSN, data loss is inescapable due to the intrinsic characteristics of WSN [32]. It is because of the sparse resources of sensor nodes [22]. Some other facets of missing data in WSN are interference of noise and environmental influence. These characteristics bring some severe issues and challenges while collecting sensor data. In this regard, the conventional data estimation method is not compatible with a wireless sensor network.

Ko et al. [33] have introduced a few WSN healthcare-related systems. In their work, they have pointed out some important technical constraints related to WSN and healthcare. One of them is related to the traversal of sensors data from one point to another; therefore, they have suggested to smartly evaluate the entire sensor data to recognize various human activities efficiently. Researchers have focused on various missing data imputation technologies. By using matrix factorization in crowd-sensing applications, authors in [34] resolved the difficulty of assessing the missing values. They proposed two unique approaches to improve the estimation accuracy by data imputation process. Gao et al. [32] introduced an algorithm based on the temporal and spatial correlation to accurately compute any missing data. In their proposed approach, they saved all the data produced at the same time. From these time-series data, the most relevant series were analyzed. They have also estimated missing values from spatial as well as temporal domains, and have added various weights.

Hong et al. [35] have explored multiple sensors in different items in a simplified kitchen scene, and determined some context-aware activities (e.g., making cold drink, making hot coffee, etc.). In this paper, the authors address the fusion process of contextual information derived from uncertain sensor data. In another work, Prabowo et al. [25] addressed the missing data handling by exploiting several machine learning algorithms on a mobile device; however, they only considered two simple classes (i.e., sitting and walking) for evaluating the performances of several algorithms to address missing data. Shahi et al. [36] presented an approach to address the problem of online activity recognition with and without unknown activities. Based on an adaptive resilient gradient algorithm, the authors proposed an online hidden condition random field (OHCRF) for human activity recognition.

Recently, low power wide area (LPWA) technology was introduced to overcome one of the limitations of wireless sensing technology, that is the restraint related to the small sensing range. LPWA technology significantly increases the sensing range with the introduction of LoRa [37]. LoRa is one of the major low-power wide-area network (LPWAN) technologies that aims to sustain connections among IoT devices over a long distance. The transmission range can be up to ten kilometers in rural areas and a few kilometers in urban areas [38,39,40,41]. The authors of [42] introduced the LoRaWAN sensors for human activity recognition. They accomplished a real experiment in a nursing care center for data collection. They have observed about 5% data missing (at a random pattern) during data collection, which concurrently reduces the recognition performance. For WSN or LPWA technology, network performance optimization is very significant for the application of healthcare domain. Kumar and Raj [43] proposed a quality of service-aware routing protocol. In this work, they sent the required sensor data with some priorities. The authors tried to avoid data sending collision for many sensors’ data at a time.

Data loss in a wireless sensor network are unavoidable because of noise, collision, and damage [26,44,45]. It has a greater impact on the quality of the collected sensor data. Missing data are either removed, or missing values are replaced by the last non-missing values; however, it may contain important information, especially for activity recognition. Nevertheless, it is not a wise idea to just remove the missing content from the dataset. There are relatively fewer works in the activity recognition domain that unambiguously addressed the problem of missing data [18,19,46]. In different areas of research, data interpolation techniques for missing data were proposed in [47,48]. Saeed et al. [49] proposed an approach for handling missing sensory features and realistic samples by adversarial autoencoder. They designed a fully-connected classification network for missing modalities. On the other hand, Pedro et al. [50] instituted four algorithms for handling missing data. In their work, they simulated various amounts of missing data. Note that the researchers mostly focused on data imputation techniques to recover missing data. Data imputation techniques are time-consuming for real settings.

There are several algorithms and techniques to estimate missing data in the domain of statistics, e.g., mean substitution, regression imputation, hot deck imputation, cold deck imputation, expectancy maximization, stochastic regression imputation, total likelihood, multiple imputations, Bayesian estimation, and pairwise and listwise deletion [51,52]; however, these algorithms are not found to be suitable for the wireless sensor network (WSN) domain due to efficiency, disregard to temporal factors, or prior requirements of missing data patterns, such as missing at random (MAR).

On the other domain, mean, median, or mode imputation only looks at the distribution of the values of the variable with missing entries. Regression imputations work well when there are correlations among the variables and missing values. In addition to model-based imputation, such as regression imputation, neighbor-based imputation (e.g., *K*-nearest neighbor (KNN) imputation) can also be explored. A KNN imputer uses the most frequent value among the closest *k* neighbors for a discrete variable, and exploits the mean or mode for a continuous variable. To explore the KNN imputation technique in a more efficient manner, it is required to train the model first with the complete data. It might be wise to use last relevant observation to fill the NA’s (NA stands for Not Available) of longitudinal data (e.g., weights of patients over a period of visits). This is called last observation carried forward (LOCF) imputation; however, while determining how to deal with missing data, it is required to understand the missing data pattern, as well as the context of the data, amount of missing data, and missing data mechanism.

During the identification of sensor data loss pattern, it was observed that sensor data missing pattern follows a random missing pattern [26,50,53]. The nature of missing data is very unpredictable. Missing patterns can be distributed at any locations in the data. The distribution of missing regions is purely random; therefore, we did not compare with any uniform random model or clumped random model. Uniform random model follows some specific time and specific missing amount. Here, ‘uniform’ implies that the missing occurrence is evenly spaced, whereas, ‘random’ indicates random spacing, and ‘clumped’ decodes that the missing data are distributed in clusters. These are not realistic random models for sensor network’s missing pattern. That is why we did not compare other random models. Hence, addressed the random missing pattern in the dataset and evaluated the performance of activity recognition having missing data. Our strategy can also reduce any overhead related to additional computational cost, processing time, and storage that is incurred while exploring the data imputation technique.

Note that there is almost no prior work that considers a realistic missing pattern in a dataset, as well as examining any missing values without imputation. Earlier, the effect of missing data situations, when missing values correspond to specific feature values, were studied. The impact of any possible good combinations of features for handling missing data for activity recognition were explored [54,55]. The randomness was varied at different levels to study the recognition results.

In this paper, we took this challenge to evaluate the performance of activity recognition without data imputation. We did not recover any missing data. In this paper, an approach is proposed to improve activity recognition performance having missing data in the dataset. To demonstrate the robustness of our approach, initially, the method was evaluated on simulated data. After obtaining good results, we explored the method on two difficult benchmark datasets.

## 3. Proposed Activity Recognition Method

**Figure 1:**
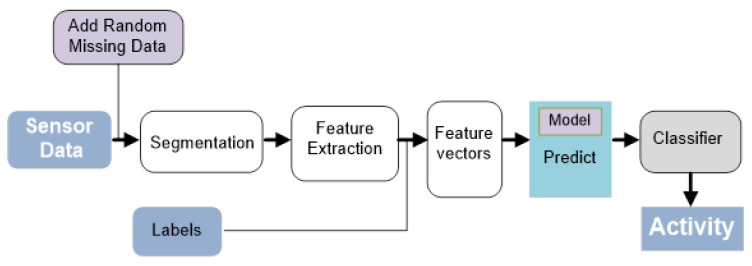
Illustration of activity recognition work flow considering missing data pattern.

Time series sensor data are required for sensor-based human activity recognition. In usual cases, the missing data issue is not considered or generated while collecting data from sensors. In this study, we addressed the missing data problem to evaluate the sensor-based activity recognition performance in a clean data environment and different percentages of missing environment. Our approach was not to recover missing sensor data, but instead, we propose a method to increase recognition performance while having different percentages of missing data by using a machine learning approach. The basic recognition process is shown in Figure 1. In this block diagram, data are collected from different sensors for different actions. Later, we extracted different statistical features from either uninterrupted or missing data at random pattern. Afterward, for activity classification, we exploited different machine learning classifiers to classify activities. In the initial dataset, there were no missing data; however, in our system, we amended the raw sensor data with different percentages of random missing data.

### 3.1. Induce Missing at Random Pattern

In this section, we explore the recognition performance in multi-sensory networks. A more realistic assumption is that missing patterns will follow the missing at random (MAR) pattern, as many sensors try to send data in real-time. Another terminology for MAR can be stated as missing conditionally at random. This missing pattern depends on some random conditions. The missing data mechanism is an important issue during the missing pattern selection. In a sensor network, missing completely at random is a straightforward situation, as it can only happen when some sensor nodes become faulty. Some replacements for the missing values can be ‘NA’, ‘None’, ‘NaN’, etc. In this work, the pattern we adopted was ‘NA’, which stands for data that are ‘not available’. Let *D* be the data from sensor, $D_{o}$ be the observed or available data, and $D_{m}$ be the data that are missing. Hence, the probability that data are missing P(r) at random can be formalized as: $P\left(rD_{o},D_{m}\right)=P\left(rD_{o}\right)$.

To induce the missing at random pattern in the dataset, we generated a random permutation of the sensor data with varying percentages and replaced those time-series tuples by ‘NA’ value. This was purely a missing at random pattern. This was a normal distribution, and there was no specific pattern or location or clustering of the missing values. Missing patterns can be distributed at any locations in the data. The random patterns were located differently, and no specific routines were followed; therefore, the distribution was purely missing at random. For creating different random missing percentages, we induced 2%, 3%, 5%, 7%, 8%, and 10% random missing data in an actual dataset. We separated each percentage of missing dataset for evaluation.

### 3.2. Methodology

A traditional machine learning model is trained and tested on the data, which has the same input feature space and the same data distribution. If the data distribution is different between train and test data, the performance of the model degrades [56]. In our approach, considering the realistic scenario, we induced missing values in both training and testing modules. In that case, the model learns with the specific percentage of missing input examples. The model initially learns the missing data behavior from the training data and it often aids for generalization. In turn, it improves the robustness of the model. So, while the model learns through having missing examples, and even tests with also missing examples, this method results in a model that will perform better when having missing data in both training and testing modules.

Therefore, we studied the effect of missing environment in the feature values [54]. Consequently, in this work, we created a missing environment in raw sensor data. We considered that missing data may occur during the period of raw sensor data collection in a practical environment. Here, we first created a simulated dataset to evaluate our approach of handling missing data. In the simulation dataset, we evaluated the performances on a clean environment. Afterward, we observed the performances in 10%, 20%, 30%, 40%, 50%, 60%, 70%, and 80% missing data on the simulated dataset.

We tested our proposed approach on two challenging benchmark datasets. These are covered in the following subsections. On the two benchmark datasets, we considered up to 10% missing data. If we chose more than 10% missing data, the recognition performance degrades drastically. After inducing the missing data pattern, we considered that the missing data were existent only in the test data module, while the training data had no missing cases. We built up a model with good quality data, and tested with the random missing data. Afterward, in our approach, in another setup, we added missing data in both training and testing sets. We studied the recognition performance—having missing data in the test module, as well as having missing data in both modules. During feature computation, an overlapping windowing approach was considered for data segmentation. The overlapping windows assist in distinguishing the smooth changeover of the signal. It also plays a significant role when the signal interval is not independent of the signals of the other intervals. It helps to cover all data information at the margin of the windows. Overlapping windows can handle the transitions more precisely. Finally, we exploited classifiers to recognize the classes smartly.

One of the most important parts of classification is to extract useful information from the sensor signal. We not explored any feature extraction method, though there are various feature extraction strategies for activity recognition. In this paper, we specifically concentrated on statistical features on time-series data. We unearthed several features. Table 1 depicts the 21 statistical features that are extracted from the rudimentary signals to generate smart features. Our study suggests that this set can profoundly assist in distinguishing distinctive activities. For the selection of important features, we considered [55], where we found that a combination of different features is useful in the extraction of useful information that has missing data. The extracted statistical features were: mean, variance, skewness, kurtosis, maximum value, minimum value, and median absolute deviation (MAD) for the *x*-axis, *y*-axis, and *z*-axis of the accelerometer data. The following paragraphs summarize the features.

Mean and variance: Mean value summarizes the data attributes for the three axes of accelerometer data. Variance is used to identify any sharp details of time series data. Consider that $X=x_{1},x_{2},x_{3},\ldots ,x_{n}$ are the time series of accelerometer data. So, the mean value is:

(1) $$\bar{X}=\frac{\sum x_{i}}{n}$$

where, *n* is the number of training instances. The variance of any random variable *X* is the expected value of the squared deviation from the mean of X:μ=E[X] and variance is:

(2) $$Var\left(X\right)=E[{\left(X-\mu \right)}^{2}].$$

Skewness: By skewness, we can measure the lack of the symmetry of the graph. It computes the skewing rate or irregularity of the probability distribution of a random variable about its mean value. For a balanced dataset, the skewness will be equal to 0. Hence, for a normal distribution, we can have a skewness of 0. It can be computed as, $\gamma =E{\left(\frac{X-\bar{X}}{\sigma^{2}}\right)}^{2}$, where $\bar{X}$ is the mean and $\sigma^{2}$ is the standard deviation.

(3) $$Skewness=\frac{n}{\left(n-1\right)\left(n-2\right)}\sum \frac{{\left(X_{i}-\bar{X}\right)}^{3}}{S^{3}}$$

Kurtosis: Kurtosis denotes the peak of a frequency distribution curve. The distribution of flatness is measured by Kurtosis. It can be defined as an average value of the variation of the time series data. It measures whether the data are peaked or flat relative to a normal distribution. It also estimates the tailedness of the probability distribution of a real-valued random variable and the degree of asymmetry of the sensor signal distribution. The peakedness or kurtosis is given by, $\mu_{4}=\sum {\left(x-\bar{x}\right)}^{k}\cdot f\left(x\right)$, where, all $\bar{x}$ are summation of values and f(x) is the probability distribution function of *x*.

(4) $$Kurtosis=\left[\frac{n\left(n+1\right)}{\left(n-1\right)\left(n-2\right)\left(n-3\right)}\sum \frac{{\left(X_{i}-\bar{X}\right)}^{4}}{S^{4}}\right]-\frac{3{\left(n-1\right)}^{2}}{\left(n-2\right)\left(n-3\right)}$$

Other features: Few other features have been extracted from the accelerometer data. For a particular signal, maxima (Max) and minima (Min) stipulate the maximum and minimum values of that signal. Moreover, the median absolute deviation (MAD) is computed as a feature. The median absolute deviation can be computed by,

(5) $$MAD=\sum \frac{x_{i}-M}{n},$$

where n= number of samples, $x_{i}=i$th sample value and M= median.

There are different classifiers and among them, we explored the support vector machine (SVM) [57] and random forest (RnF) [58] methods. As sensor-based data are not computationally-expensive, similar to the image or video data, we concluded that SVM or RnF classifier can perform without much processing time. For SVM, we exploited the radial basis function (RBF) as the kernel function. SVM is very well-known for its strength in classifying many binary or multiclass scenarios in computer vision, imaging, and other arenas. SVM has been employed in several thousand good research so far.

Apart from the SVM, the random forest classifier is another important classifier that is also widely explored by the researchers. The RnF’s strength is its ability in classifying multiclass scenarios with a regression algorithm [59]. The random forest classifier has a set of basic classifiers of decision trees, which are produced in a random manner according to the sampled data in training dataset [59]. These decision trees are trained autonomously. In the test module, each class label is developed based on the multiple classifiers’ prediction levels [60]. According to the study in [60], the most inconsistency-tolerant classifier is the random forests; therefore, if the missing rate is higher, it is supposed to perform reasonably better than a basic decision tree, Bayesian network, logistic regression, K-means, and K-nearest neighbors (KNN) algorithm [60]. For Random forest, we implemented R’s approach based on the Breiman’s random forest algorithm. In the implementation, there were 500 trees. This number was set to a relatively high number so that every input row can be predicted at least a few times.

## 4. Experimental Results

In this research work, we explored two benchmark datasets to evaluate the method: these were the human activity sensing consortium (HASC) dataset [27], and the single chest-mounted accelerometer (SCMA) dataset [28]. The HASC dataset comprises more than 6700 data, taken from the accelerometer. These were captured from 540 subjects. This is one of the few and largest datasets in terms of the number of subjects that were engaged to collect accelerometer-based dataset [61]. Usually, most of the datasets were created using 10 or 20 subjects. Hence, this dataset is indeed a difficult one to challenge. There are six activity classes—stay in a normal position, walking on the floor, jogging, skipping action, walking up the stairs, and walking down the stairs. The data were collected through two different modes: (i) segmented dataset: segmented data per activity per person; (ii) sequential dataset: several activities in a sequence by a person. The latter mode (i.e., continuous and multiple activities) makes this dataset even more challenging. Apart from the multiple activities in a sequence, the activities were taken at a random order as well. Usually, each activity was taken for 10 s or above for the sequential dataset. The segmented data were measured for 20 s and in *.CSV format. Conventionally, the segmented dataset was considered as a training module, whereas the sequential dataset was taken as a testing module. The sampling frequency was 10 to 100 Hz; the age range of all young subjects was 21 to 32 years. The leave-one-person-out cross-validation pattern was considered in our research.

On the other hand, the single chest-mounted accelerometer [28] dataset (single chest dataset) was designed from 15 subjects who put the wearable accelerometers on their chests. There are seven activity classes in this dataset—standing; walking normally; working at a computer; standing-up, walking and going up or down stairs (a combined and complex activity); walking up or down a staircase; talking while standing; and walking and talking with someone. Though there is are relatively few subjects in this dataset, the activities are complex and challenging when compared to many other existing datasets. The sampling rate of each wearable accelerometer sensor was 52 Hz. Similar to the HASC dataset, the data were stored in a *.CSV format. Before explaining our results, we present the missing data concept in relation to our simulated dataset.

### 4.1. Results for the Simulated Data

**Figure 2:**
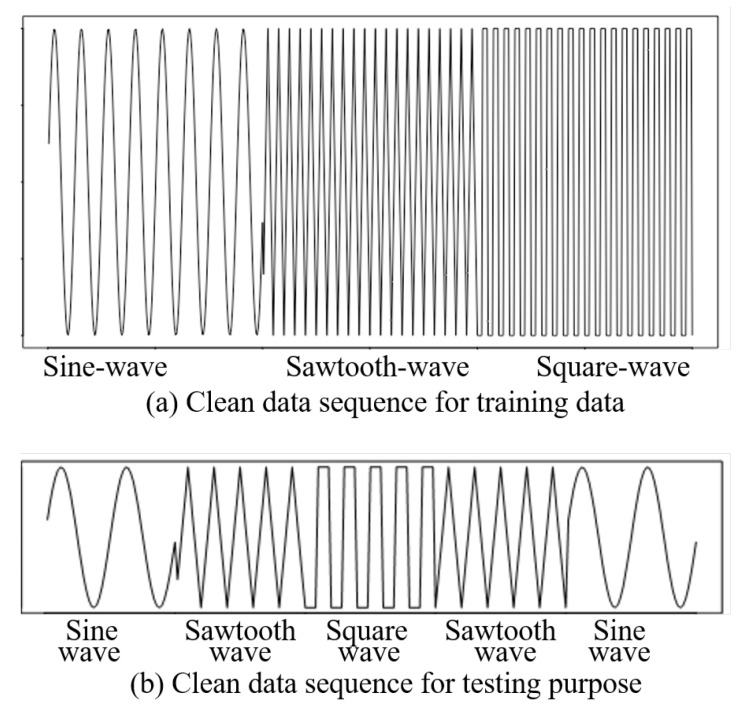
Time-series data having three different signals: sine wave, sawtooth wave, and square wave; (**a**) clean data sequence for training data; (**b**) clean data sequence for testing purposes.

In this simulated dataset, we introduced three patterns of signals or data—sine wave, sawtooth wave, and square wave. There were 1200 data entries in the simulated dataset for training and testing. Each data pattern contained 400 entries separately. Initially, these are continuous time-series data, and later, we added some random missing patterns to evaluate our method. This is done to justify or confirm the classification accuracy. At the beginning, we took continuous time-series data for the three signals (Figure 2). The training data had three sequences (top image in Figure 2). The bottom image of Figure 2 has different five signals that were considered as the testing data. Then, we added some missing signals randomly in the sequence—both for training data and testing data. The added missing data were considered from 10% to 80%. However, up to 60% missing data in this short sequence was found to be reasonable. After that, the missing data rate was extremely high and hence, the recognition result fell drastically. The recognition results were computed for clean data as well as for different levels of missing data patterns (from 10% to 80%). These were accomplished when the training data were clean but the testing data had missing data in a random order.

**Figure 3:**
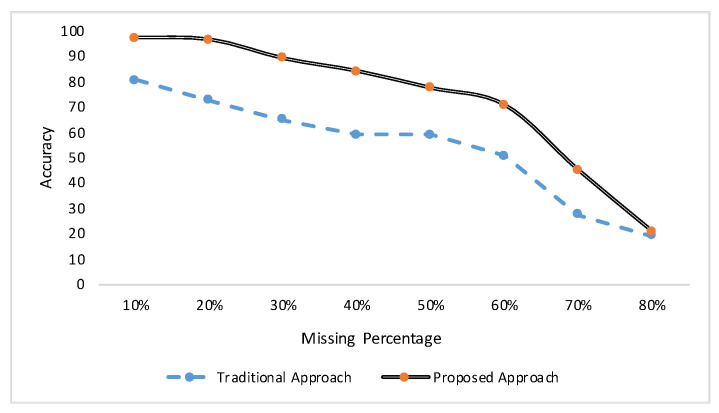
Recognition results for the simulated data with different data loss rates (from 10% to 80%). Traditional approach represents the results when the training data were clean while the testing data had missing patterns. Proposed approach demonstrates the results when both data had missing patterns.

As mentioned above, we computed the recognition rate when the missing data were present in both training and testing data. These results are demonstrated in Figure 3. It is evident from these results that by using our proposed approach when both training data and testing data have missing data patterns, the recognition results are always enhancing. According to our study, we have found that when there is no missing data, the recognition rate is 100% in this simulated data [55]. However, after adding missing data randomly in the sequences for testing data only, as well as, for both training and testing data—we can have a better recognition rate when both training and testing data have missing patterns randomly.

### 4.2. Results for the Benchmark Datasets

In this paper, missing datasets were augmented as per the following three conditions on the two benchmark datasets (HASC dataset and the single chest dataset): (i) activity classification without any missing data; (ii) activity classification with missing data in the testing part; (iii) activity classification with missing data in both training and testing parts.

**Figure 4:**
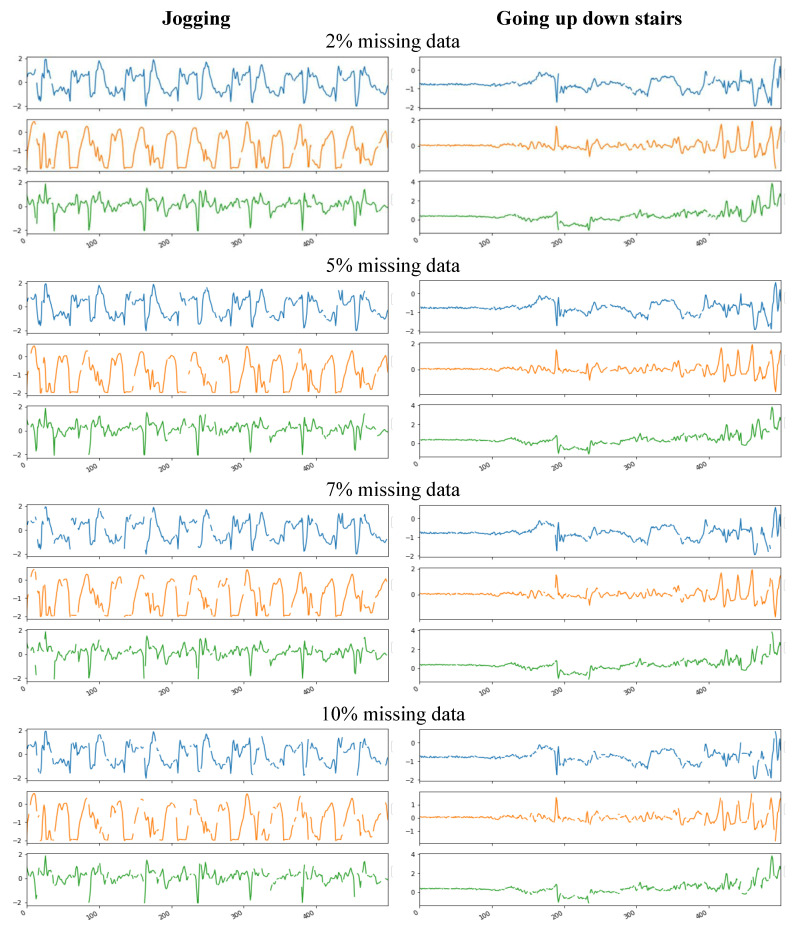
Data sequence of ’jogging’ and ’going up down stairs’ from two benchmark datasets that have different percentages of missing data. Missing rates are from 2% to 10%. Accelerometer data for *x*-, *y*-, and *z*-axes are shown for each case.

To demonstrate the variations and data loss patterns, we depicted two different actions and realistic missing patterns in Figure 4. In Figure 4, data sequences of two activity classes from the two datasets were demonstrated. Among the two datasets, the single chest dataset is an imbalance dataset in comparison to the relatively fairly-balanced HASC dataset. This shows how missing data were distributed among each activity class for different percentages of missing data. For missing data classification, we considered the missing at random pattern, which is a more realistic situation during real field data collection. We generated a random permutation of the sensor data with varied percentages. In this scheme, there was no specific patterns or locations or clustering. It is the sensible scenario while assembling sensor data from multiple sensors; missing data may occur at random patterns, which can be distributed at any location in the data. We embraced two activity classes: ‘jogging’ and ‘going up down stairs’ from two benchmark datasets to illustrate the distributions of data missing in various percentages of missing data. Missing rates were from 2% to 10%. Accelerometer data for *x*-, *y*-, and *z*-axes are shown for each case. In the real dataset, it was not considerable to have missing data for more than 8% to 10% in the context of activity recognition.

**Figure 5:**
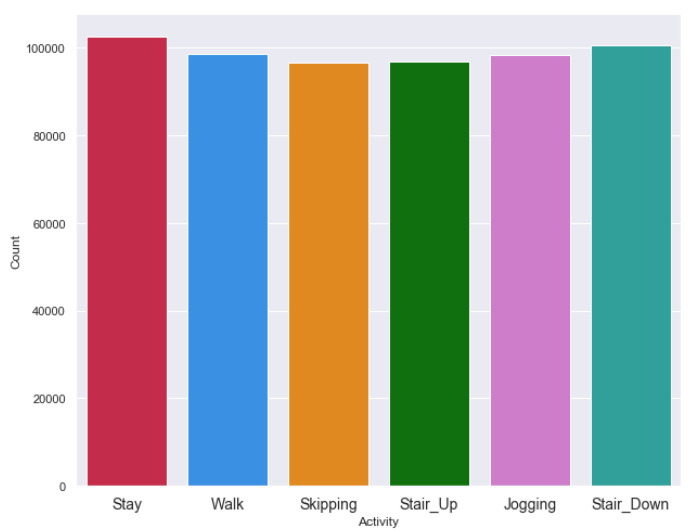
Activity record count in HASC clean dataset.

**Figure 6:**
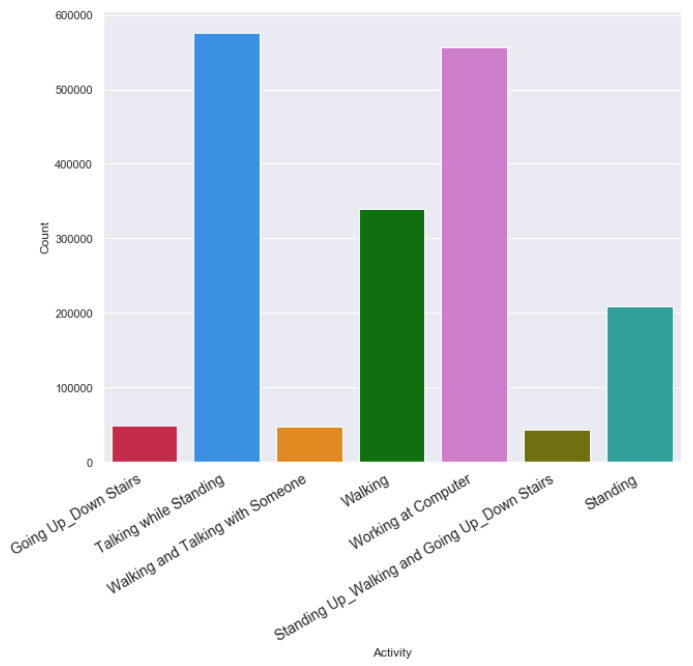
Activity record count in Single Chest clean dataset.

Figure 5 and Figure 6 exhibit the activity counts of HASC and single chest dataset. HASC dataset has regular activity classes, whereas, the single chest dataset has two overlapping activities for each class. For example, the going up and down stairs together comprise as one class; whereas, the standing up-walking and going-up-down stairs combine as another class. We considered two datasets so that we can evaluate activity recognition performance having missing data in straightforward activity classes, as well as on several overlapping activity classes.

**Figure 7:**
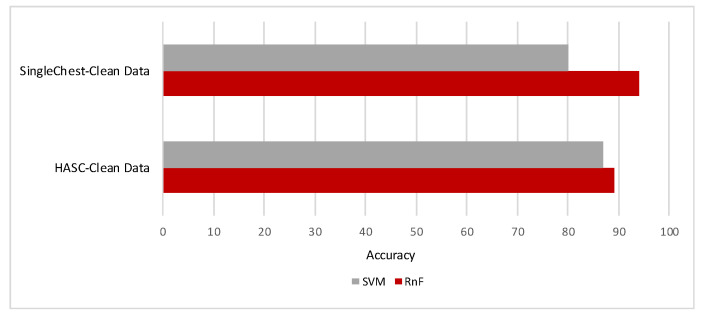
Recognition results for clean data for both datasets (in %).

**Figure 8:**
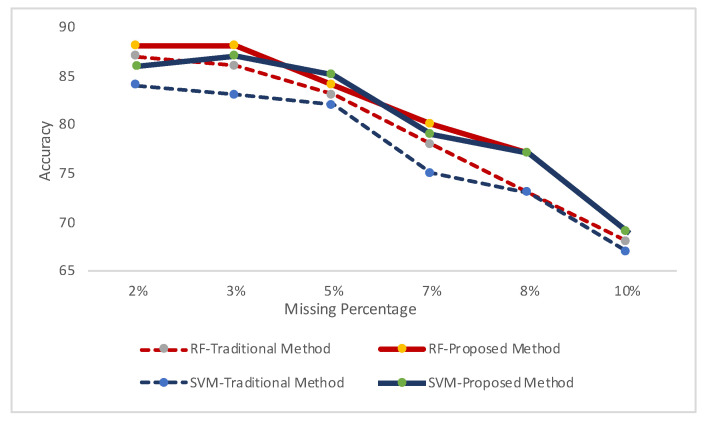
Missing data analysis for activities of human activity sensing consortium (HASC) dataset.

**Figure 9:**
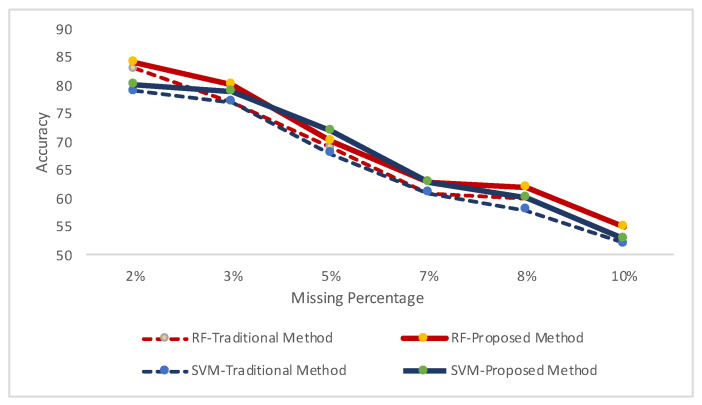
Missing data analysis for some activities of single chest dataset.

We have orchestrated different missing percentages of data randomly in these datasets. Then, we have observed the performances of recognition results in the following three environments:

- (Case-i) clean data for both train and test module (Figure 7);
- (Case-ii) clean train data and missing data in test module (Figure 8 and Figure 9); and
- (Case-iii) missing data in both test and train modules (Figure 8 and Figure 9).

In Figure 7, we have demonstrated the recognition results for clean datasets (Case-i) in two classifiers. Random Forest classifier has performed better recognition results for both cases. In Figure 8 and Figure 9, we demonstrated the recognition results for both datasets on different levels of missing data in two environments: clean train data and missing data in test module (Case-ii); and missing data in both test and train modules (Case-iii). We accomplished these to demonstrate whether our simulated analyses have correlation with real datasets.

In Figure 8, the results are showing for HASC datasets, for both classifiers, in two environments: having missing data rate from 2% to 10%. More missing data produces lower recognition results, hence these are unacceptable. Hence, we have demonstrated the results up to 10% missing data rate. For the both classifiers, we have found better recognition rates by using our proposed approach when the both train and test datasets have missing information. In the similar fashion, Figure 9 demonstrates the approval of the above conclusions that our approach can produce higher recognition results when the both train and test data have missing patterns.

### 4.3. Effect of Different Window Sizes for Different Missing Rates

**Figure 10:**
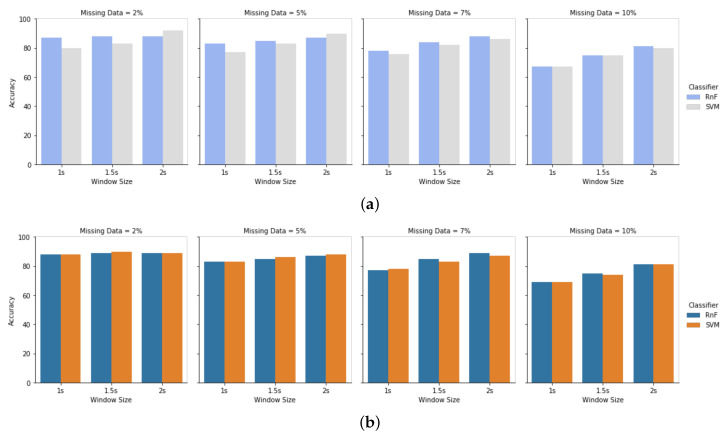
Evaluation of the HASC dataset for window size 1 s, 1.5 s, and 2 s. Missing percentage 2%, 5%, 7%, and 10% in two scenarios: (**a**) HASC dataset for the traditional approach; (**b**) HASC dataset for the proposed approach.

**Figure 11:**
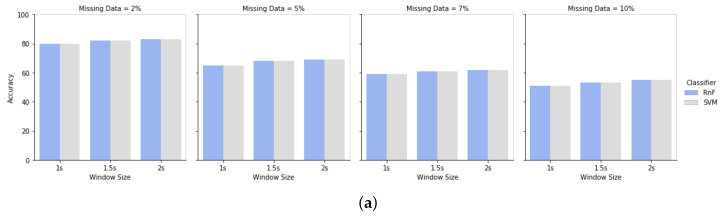

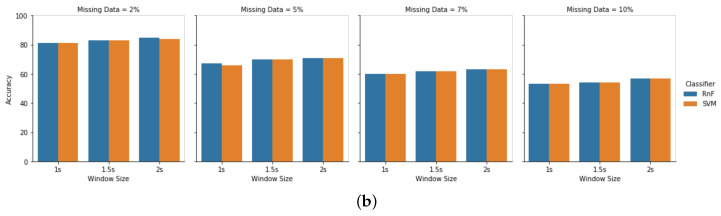
Evaluation of the single chest dataset for window size 1 s, 1.5 s, and 2 s. Missing percentage 2%, 5%, 7%, and 10% in two scenarios: (**a**) single Chest dataset for the traditional approach; (**b**) single chest dataset for the proposed approach.

Window size and window sliding widths have impacts on handling missing data in the dataset. Figure 10a is the result of having missing data in the test module, while train data was clean in HASC dataset. We have evaluated the results for the window sizes 1 s, 1.5 s, and 2 s, having missing percentages of 2%, 5%, 7%, and 10%. Results demonstrate that the window size of 2 s has less impact of missing information rather than the window size of 1s. It is because of the fact that a larger window size contains more information of a particular activity class. When any specific activity class is affected more by the presence of missing data, it can handle missing information with a larger window size. On the contrary, a smaller window size may lead to a decrease in the accuracy of not having enough activity class information in that particular window period. Figure 10b shows the result of our proposed approach of having missing data in both train and test modules in window size 1 s, 1.5 s, and 2 s for the HASC dataset. It has shown that using the proposed approach of handling missing data in both train and test modules can increase recognition performance. Figure 11 demonstrates the same evaluation for the Single Chest dataset.

## 5. Analysis and Discussion

### 5.1. Analysis for Synthetic and Experimental Dataset

The simulated data demonstrated some evaluations based on mean and variance. With simulated data, it shows recognition performance as 100%, when it has no missing data in the dataset. Afterward, we evaluated the performance of our approach by adding different percentages of random missing data (from 10% to 80%) in the simulated dataset. Regarding the missing data % variations in simulated data and on benchmark datasets, we would like to mention that the simulated data is a trivial dataset to test the model and to get feedback—based on which, we can work further; therefore, we created a higher % of missing data. The patterns are simple in the simulated dataset, therefore, we need to add more missing data. On the other hand, for real-life situation in wireless sensor network, the missing data are not of a higher rate. In reality, if more than 10% data are missing, for a sequence to decode various activities, there is a huge chance that one or more activities can be completely ignored or missed. Usually, in WSN, missing data at random occur, which is why we explored this strategy. Missing data for a large chunk of data means either the sensor network has experienced a major technical flaw, or there was an error in data transmission. Those issues can be handled by network administrators or network-based data loss assessment mechanism. We ascertain that the current work has successfully dealt with the missing data scenario in a realistic manner.

In our approach, we observed that when both modules have missing data, it always demonstrated better performance compared to the cases of having missing data in only during the testing time. With both HASC and single chest datasets, the random forest classifier provided better recognition results than the support vector machine classifier for both datasets when data are fully clean. With the HASC dataset, it is 89% and 87% with random forest and support vector machine classifiers, respectively. When we have evaluated the same evaluation with the single chest dataset, it achieved 94% and 80% by random forest and support vector machine classifiers, respectively. Evaluation with clean datasets depicted better performances by the random forest classifier. Sensor-based data are not computationally-expensive like image or video data; therefore, we considered that both classifiers can perform without much processing time.

In the evaluation time with HASC dataset using the random forest classifier and a combination of 21 features, we achieved a better result of handling different levels of missing data in the dataset. When a dataset has 3% missing data in the test module, we found that the accuracy is 86%. On the other hand, it improved the performance to 88% when both modules have missing data. In the single chest dataset, the performances of random forest and support vector machine had almost similar results during the handling of different percentages of missing data. With 3% of missing data in the test module, it showed a 77% recognition accuracy, but when both modules have missing data, it improved to 80%. The evaluation results demonstrated consistently better performance when we trained the dataset with a missing data environment, and then evaluated with missing data environment at the same time. It has improved the performance compared to the only missing data in the test dataset; however, this enhanced recognition result is not significant. In terms of accuracy and having missing data, we identified that the HASC dataset’s performance was superior, because the HASC dataset activity classes do not much vary that much in terms of complexity compared to the single chest dataset.

**Figure 12:**
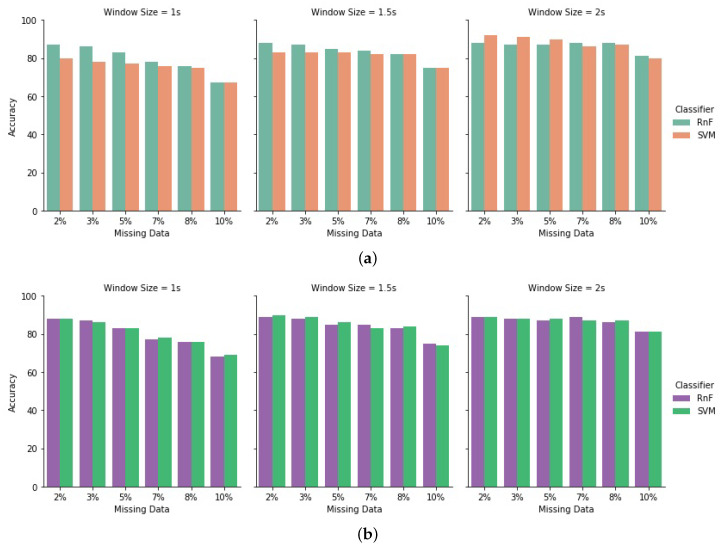
Evaluation of HASC dataset for window size of 1 s, 1.5 s, and 2 s; and missing percentage of 2%, 3%, 5%, 7%, 8%, and 10% in two scenarios: (**a**) HASC dataset on traditional approach; (**b**) HASC dataset on proposed approach.

We tested in different percentages of missing data levels. Table 2 represents the comparative result analysis of 5% and 8% missing data in both datasets. We observed that when we added even 5% missing data in the testing module, the recognition rate became 83% by using RnF in HASC dataset, whereas it lowered to 69% in the single chest dataset when only the test module had missing data. By using the SVM classifier too, it was 82% in the HASC dataset, but 68% in the single chest dataset: even the recognition performance in single chest dataset reduced to 60%, when 8% random missing data were present in the test dataset. Performances always improved slightly when using our proposed method when both training and testing modules had missing data in both datasets. On the other hand, for the HASC dataset it was 73% when using RnF and SVM. We evaluated the performances with different window sizes, both datasets performed well with a higher window size in any missing percentages. We evaluated different missing percentages (e.g., 2%, 3%, 5%, 7%, 8%, and 10%) for different window sizes (i.e., 1 s, 1.5 s, and 2 s)—as demonstrated in Figure 12. We observed that the window time of 2 s has performed well in any missing percentage situations. The performances improved for having missing data in both the testing and training modules. We can state that the amount of data is an important issue to estimate the correct activity labels even having a missing data environment. Finally, we noticed that the fairly balanced HASC dataset can handle missing data in a considerable manner, compared to the single chest dataset, which is a more imbalanced dataset. We chose these two different, but genuinely challenging, datasets to manifest the validity and robustness of our approach.

In our approach, the model learns with the specific percentage of missing input examples, and their associated outputs also have different percentages of missing data. In this way, the model initially learns the missing data behavior from the training data. In this study, we explicitly induced different percentages of missing data randomly in the raw sensor data to train the model with missing data. Learning with missing data reinforces the model to regulate missing data during the classification of various activities while having missing data in the test module. However, this method, under realistic circumstances (i.e., real data loss issues in sensor network) where the distribution of the inputs are different between source and target data, approximates the training and testing data distribution totally differently due to random loss happened in training and testing in unknown pattern. Traditional machine learning model is trained and tested on the data, which has same input feature space and same data distribution. If the data distribution is different between train and test data, the performance of the model drops. This approach manifests the plausibility of machine learning model, while it can learn and predict from the identical domain.

### 5.2. Realistic Settings Data Analysis from Real Nursing Care Data

We demonstrated that the approach performs well on the synthetic data and experimental data. Note that sensor-based human activity recognition approaches are still based on limited datasets, by 6 to 10 classes on average, accomplished by few subjects only without any real-life challenging situations, let alone having missing data. Activity recognition based on sensor data is the prominent research area in the ubiquitous computing community. However, there are a very few examples of real-world activity recognition at hospital and nursing homes. Therefore, it is really a daunting task to develop more realistic datasets as well as, explore various methods. However, this is the goal of the researchers to achieve. With this reality, we explored our experiments on two really challenging datasets.

**Figure 13:**
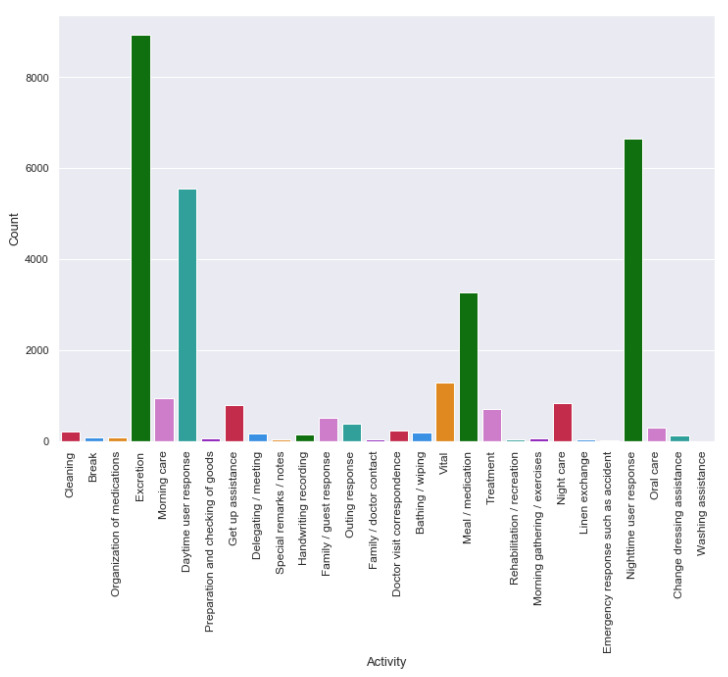
Nursing care dataset with 21 complex activity classes.

Nevertheless, we mention one of our datasets that is explored in a realistic setting on a real-field nursing care dataset [15]. We present this information to show that even in real-life, missing data in sensors are mostly random in nature, which we have explored in this work. In the paper [15], we introduce a system to integrate activity recognition into nursing care record system. The system exploits smartphones to create nurse work and care records, and explores a cloud service to collect sensor data for activity recognition in a real nursing care center for four months. During this experiment, 38,076 activity labels, 2834 h of sensor data, and 46,803 care details were collected. Figure 13 shows that this dataset contains 28 complex nursing care activity classes for regular nursing care support, which was challenging to collect, such as vitals (checking), excretion, bathing/wiping, etc. To collect nursing care residents’ complex activity data, 27 people, including 23 caregivers and 4 nurses, conducted this experiment in a real nursing care facility center. It required capturing continuous sensor data by using mobile sensors to capture nursing activities; however, there is still a challenge due to the missing samples.

**Figure 14:**
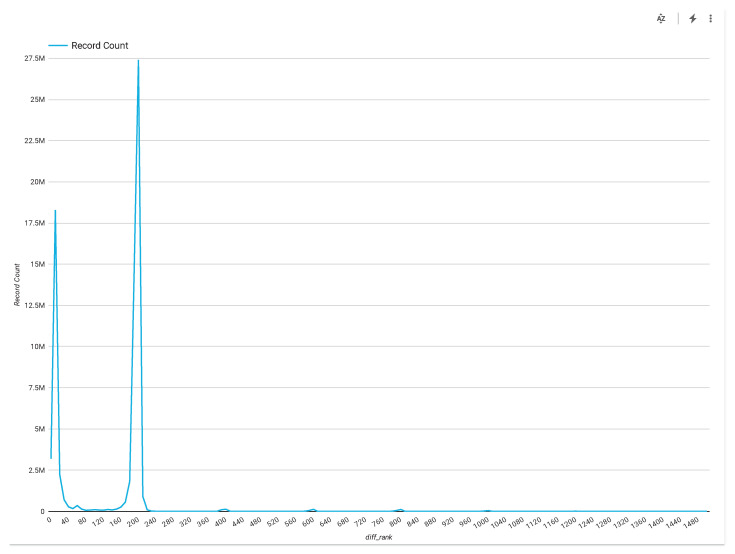
Demonstration of samples analysis from a real-life nursing care data (*x*-axis is the timestamp difference for number of samples received in millisecond vs. record count).

To evaluate the missing pattern from this dataset, we depict Figure 14. Here, the abscissa represents the delay or difference of timestamp for receiving samples in millisecond, whereas, the ordinate denotes the record count. In the case of no mission data in the dataset means 200 ms sampling rate for all data. In that case the graph will be one spike in 200 ms sampling rate for all data. If there are no missing data and no variance in the delay then it becomes like one single spike for all data received in 200 ms sampling rate. It is the ideal situation. If we consider the missing value then there will be variance in the 200 ms sampling time but in this time there is small variance in 200 ms sampling time and it is because of jitter. If we have large variance in 200 ms sampling time, then this graph become wider at pick of 200 ms timestamp. When we have one sample missing then it means next sampling time will be 400 ms as 200 ms timestamp is the ideal case for data collection. We have a sample at timestamp 200 ms (this is the absolute time), then 400 ms timestamp as well as 600, 800, and 1000 ms (this carries missing samples information). If one sample is missing at 200 ms timestamp then we can get a record count information in 400 ms or if two samples are missing then we can get missing sample information at the 600 ms timestamp.

Consider the analysis in Figure 14, which shows that at 200 ms, samples can be picked, which also have around 1–5 ms. There is no large variance in the 200 ms time. The graph is like a spike graph for this case as all time difference are 200 ms and it is the top sampling timestamp during data collection. We have this kind of small samples because we can see the some of the pick at the very small number/small difference (400, 600, and 800 ms). If we observe the 400 ms sampling data amount then we can observe small number of missing samples and if we observe 600 ms sampling data amount then it means that continuously two succeeding samples are missed. Sometimes this missing pattern are random at any timestamp and sometimes it is continuously missing in two succeeding samples. So, we can see from realistic situation that randomly and continuous missing happens during any consecutive timestamp in the practical application.

To count the missing ratio, we found that missing ration between 400 and 600 ms is 1.15% and missing ratio between 600 and 800 ms is 1.27%, while it is 0.54% during 800 and 1000 ms sampling duration. It is not a large missing ratio in this real application setting. The reason is that our entire networking system and battery lives of sensors were properly checked and enriched with the best-possible manner, so that no missing data can happen. However, in real-life, the causes of missing data may happen and we can face more challenges. After this realistic dataset analysis for missing pattern and missing sample ratio, we can ascertain that our proposed approach can be applicable for realistic settings as well to improve activity recognition. If the missing pattern is random and the missing ratio is approximately 2%, then using our proposed approach, we achieved enriched accuracy up to 88% (for the HASC dataset) and 84% (for the single chest dataset) by using the RnF classifier, and 86% and 80% for these datasets, respectively, by exploiting SVM classifier.

## 6. Conclusions and Future Scopes

Human activity recognition is a very important research area. In this work, we explored the human activity recognition based on wearable sensors and we addressed a very important challenge, that is, the presence of missing data in the activity recognition without data imputation. We rigorously studied the missing data issue in this domain and proposed an approach to handle the missing data. In our method, we explored different types of twenty-one statistical features that were computed for the three axes of the accelerometer. The features are: minimum value, maximum value, mean, median absolute deviation, variance, skewness, and kurtosis. These features are fed into a smart classifier to recognize different activity classes. Explored classifiers are random forest and support vector Machine. We created various scenarios for experimentation, having various levels of missing data in a random manner, as well as creating different window sizes with varying sliding widths in the time-series data.

Initially, we developed three simulation data patterns with and without any missing data. Based on the successful analysis, we then explored activities from two very challenging benchmark datasets for our experiment. These are the HASC dataset and the single chest dataset. Under various experiments of random missing data levels, we recognized activity classes. From our experimental analysis, we can conclude that the recognition results improve by our proposed method when missing data patterns are available in both training and testing modules without recovering the raw data.

In the future, it is required to explore for more features to enhance the recognition results in the case of missing data at random order; however, modeling of the realistic nature of missing data under all major circumstances can be explored by network-based researchers. This can be a rudimentary step so that, based on any realistic model, we can explore human activity recognition in genuine nursing home or healthcare facilities, where missing data are more prevalent. Smarter datasets are required having realistic missing data from various sensors and there explorations.

## Figures and Tables

**Table 1:** Extracted features on the HASC and Single Chest datasets.

1. Mean-X	2. Mean-Y	3. Mean-Z
4. Variance-X	5. Variance-Y	6. Variance-Z
7. Skewness-X	8. Skewness-Y	9. Skewness-Z
10. Kurtosis-X	11. Kurtosis-Y	12. Kurtosis-Z
13. Max-X	14. Max-Y	15. Max-Z
16. Min-X	17. Min-Y	18. Min-Z
19. Median-X	20. Median-Y	21. Median-Z

**Table 2:** Result analysis having 5% and 8% Missing data in both datasets.

	HASC Dataset RnF		HASC Dataset SVM		Single Chest Dataset RnF		Single Chest Dataset SVM	
	5% Missing Data	8% Missing Data	5% Missing Data	8% Missing Data	5% Missing Data	8% Missing Data	5% Missing Data	8% Missing Data
Traditional Method	83%	73%	82%	73%	69%	60%	68%	58%
Proposed Method	84%	77%	85%	77%	70%	62%	72%	60%
